# Feasibility of a Supplemental Phonological Awareness Intervention via Telepractice for Children with Hearing Loss: A Preliminary Study

**DOI:** 10.5195/ijt.2017.6216

**Published:** 2017-06-29

**Authors:** SUE ANN S. LEE, BRITTANY HALL, SHERRY SANCIBRIAN

**Affiliations:** 1 TEXAS TECH UNIVERSITY HEALTH SCIENCES CENTER, LUBBOCK TEXAS, USA

**Keywords:** Children, Hearing loss, Intervention, Phonological awareness, Telepractice

## Abstract

The goal of the current study was to examine the feasibility of a telepractice intervention to improve phonological awareness skills in children with hearing loss as compared to a conventional in-person intervention. Twenty children with hearing loss participated in this study. Two groups of ten children each received a supplemental phonological awareness intervention either via telepractice or an in-person service delivery model. Within each of the two groups, five children were enrolled in preschool or kindergarten and five children were enrolled in first or second grade. The two groups of children demonstrated similar phonological awareness, non-verbal IQ, and vocabulary skills during pre-tests. After a 12-week intervention children with hearing loss showed improved phonological awareness skills as measured by a standardized post-test. No significant differences were found between the performance of the telepractice group and in-person group. Nor was a significant interaction found between the two age groups (PreK/K vs. 1st /2nd grade) and the two types of service delivery models (in-person vs. telepractice). The results suggest that a telepractice service delivery model is feasible for young children with hearing loss, and that telepractice may be as effective as in-person intervention in improving phonological awareness skills.

Recent advances in information technology allow speech-language pathologists (SLPs) and audiologists to evaluate and treat patients with communication disorders via telepractice. The American Speech-Language-Hearing Association (ASHA, 2005), defines telepractice as the application of telecommunication technology for SLPs and audiologists to deliver assessment, intervention, and/or consultation services to patients at a distance.

While telemedicine and telehealth have been well documented as service delivery models in medicine, there has been less written about the use of telepractice to deliver speech-language pathology services. Ekeland, Bowes, and Flottorp (2010) examined multiple systematic reviews of the impact of telemedicine services. They studied 80 systematic reviews on telemedicine including 1593 individual articles. In contrast, Mashima and Doarn’s (2008) and Reynolds et al.’s (2009) systematic reviews of telepractice for SLP services included only 40 and 62 articles respectively, reporting that few studies provided a high level of evidence. Recently, Coleman et al. (2015) conducted a systematic review of 218 studies on assessment and intervention via telepractice for cognition and communication skills in adults with traumatic brain injury. They reported that most studies examined adults, with fewer including children. Only a small number of quasi-experimental or experimental studies of telepractice were available. Thus, it is critical to evaluate whether intervention via telepractice is feasible for children with speech and hearing disorders.

While telepractice has been proposed as a viable service delivery model to provide SLP and audiology services to children with hearing loss (Cohn & Cason, 2012), the majority of research with this population is currently limited to the provision of audiology services. There is little evidence on the effectiveness of SLP service via telepractice for children with hearing loss. Edwards, Stredler-Brown, and Houston (2010) summarized existing research regarding telepractice for children with hearing loss. Inclusive of both the pediatric and adult populations, they located only nine telepractice studies for audiological assessment and three studies for audiological treatment. No study provided telepractice SLP services for children with hearing loss.

Recently, however, a few studies using telepractice to deliver SLP services for children with hearing loss were reported. These focus on the early intervention setting with families of infants and toddlers with hearing loss. Houston (2011) reported on a small pilot study of early intervention via telepractice for families of infants with hearing loss. Behl, Callow-Heusser, and White (2013) conducted a randomized control study of 27 families of infants and toddlers with hearing loss and evaluated pre- and post-test measurements on child and family outcomes, family and provider satisfaction, and cost. Most recently, Constantinescu et al. (2014) provided auditory-verbal therapy (AVT) via telepractice to seven young children (mean age 2:4 years) with hearing loss. The children’s outcomes using the Preschool Language Scale-4 (PLS-4) were compared with another group of seven age equivalent children with hearing loss who received AVT in the traditional in-person method. There were no significant differences in total language, auditory comprehension, and expressive communication subtests between the two groups. These studies reported that telepractice may be a useful service delivery model for children with hearing loss in early intervention settings as compared to an in-person delivery method.

Telepractice is a necessary service delivery model for children with hearing loss because in-person professional services are limited due to the lack of qualified service providers (Houston, 2011). A number of researchers and practitioners (Besculides, Saltzman, Ireys, & White, 2010; Houston, Munoz & Bradham, 2011; Joint Committee on Infant Hearing, 2007) raised similar concerns about the shortage of qualified professionals for children with hearing loss. Telepractice would be a way to resolve this issue. Therefore, more research should be conducted to support the benefits of the telepractice service delivery model for this population.

## PHONOLOGICAL AWARENESS IN CHILDREN WITH HEARING LOSS

Phonological awareness refers to “the metalinguistic skills involved in understanding that spoken words can be broken down into smaller parts” (Gillon, 2004, p.11). The current study examined phonological awareness skills for children with hearing loss because individuals with hearing loss demonstrate significantly lower reading skills when compared to individuals with normal hearing. On average, high-school graduates with profound hearing loss had a third- to fourth-grade reading level (Krose et al., 1986). Geers and Hayes (2011) also reported that 53% of high school students with hearing loss scored more than one standard deviation below the mean on a standardized reading test, a proportion that was three times greater than that of their counterparts with normal hearing. Research shows that phonological awareness skills are an important prerequisite for later reading development in children with and without hearing loss (Harris & Beech, 1998; Stahl & Murray, 1994). Webb and Lederberg (2015) suggest that phonological awareness training is necessary for children with hearing loss to help them develop their literacy skills.

Phonological awareness skills are taught in kindergarten and elementary school classrooms in the US throughout the course of literacy development. The Texas Essential Knowledge and Skills (TEKS, 2017), the Texas state standards for students’ knowledge and skills at each grade level, suggests that various phonological awareness skills should be taught in the classroom. In particular, phonological awareness skills are targeted during kindergarten and early first grade to support children’s development of the requisite literacy skills to learn to read. According to the TEKS, children enrolled in kindergarten are expected to develop concepts of print, the relationship between letters and sounds, and the identification of syllables and rhymes. They are also expected to segment and blend syllables, segment and blend onset and rhyme, isolate initial sounds in one syllable words, and segment one syllable words into two or three phonemes. During first grade, children should be able to generate rhyming words, add/delete/substitute a sound in words, blend sounds into words, isolate initial, medial, and final sounds in one syllable words, and segment one syllable words into three to five phonemes. After achieving these phonological awareness skills, second grade students are able to achieve more complex phonological awareness skills such as decoding consonant blends, digraphs, vowel digraphs and diphthongs.

Children with hearing loss enrolled in US schools are educated with a reading curricula similar to that developed for children without hearing loss. Additionally, phonological awareness instruction is often provided with visual support such as Cued Speech or Visual Phonetics (Colin, Magnan, Ecalle, & Leybaert, 2007; Narr, 2008; Trezek, Wang, Woods, Gampp, & Paul, 2007). Even though children with hearing loss receive phonological awareness instruction in the classroom, research typically shows that they demonstrate delayed phonological awareness regardless of the degree of hearing loss (Kyle & Harris, 2011; Miller, 1997; Moeller et al., 2007; Most, Aram, & Andorn, 2006; Sterne & Goswami, 2000). Therefore, a supplementary phonological awareness intervention should be beneficial to children with hearing loss. This might be delivered by a speech-language pathologist or another professional familiar with the development of these skills.

Currently, few studies examine the effects of phonological awareness training for children with hearing loss. Entwisle, Brouwer, Hanson, and Messersmith conducted a systematic review of emergent literacy interventions for preschool and kindergarten aged children with hearing loss. They had only three studies (Aram, Most, & Mayafit, 2006; Miller, Lederberg, & Easterbrooks, 2013; Smith & Wang, 2010) that fit the inclusion criteria. However, the Aram et al. study was not an intervention study; it examined correlates of mother-child interaction with literacy development in children with hearing loss. We found two additional studies (Syverud, Guardino, & Seiznick, 2009; Werfel & Schuele, 2014) in which phonological awareness intervention was provided for children with hearing loss.

Syverud et al. (2009) investigated the effectiveness of a phonological awareness curriculum, *Teach Your Child to Read in 100 Easy Lessons* (Engelmann, Haddox, & Bruner, 1983) as used with a first grade deaf child. They reported that the child’s phonological decoding skills improved after 8 weeks of intervention. Smith and Wang (2010) examined the impact of a 6-week intervention using Visual Phonics on the phonological awareness and speech production of a 4-year-old deaf child and found that Visual Phonics significantly increased phonological awareness and speech production. Werfel and Schuele (2014) also investigated whether phonological awareness training would result in increased initial sound segmentation skills in two preschool children with severe to profound hearing loss. They used a single subject multiple baseline design across three behaviors (initial phoneme /m/, /d/, /b/ identification). The authors concluded that initial phoneme awareness training led to an increase in initial sound segmentation skill, though consistent performance was not observed during the maintenance period. These studies examined only a small number of children (i.e., one or two children).

Miller et al. (2013) study included five children whose ages ranged from 3:8 (i.e., 3 years 8 months) to 5:11 with hearing loss. They provided one hour of phonological awareness instruction, four days a week for four to six weeks. Miller and colleagues deployed a single subject multiple baseline design across three behaviors (syllable segmentation, initial phoneme isolation, and rhyme recognition) and found that four children showed an average increase of 66% in syllable segmentation skills, a 68% in initial phoneme isolation, and 75% in rhyme discrimination.

Because the previous studies employed single subject designs, their findings are difficult to generalize. Well-designed experimental studies with a larger number of children with hearing loss are needed to investigate the effects of phonological awareness intervention. Therefore, the current study employed a larger number of children with hearing loss and examined whether phonological awareness intervention leads to improvement in phonological awareness skills in children with hearing loss.

## METHOD

### PARTICIPANTS

Twenty children with hearing loss enrolled in local schools in West Texas participated in this study. They were referred by SLPs or itinerant teachers of Regional Day School Program for the Deaf. The children met the following criteria: 1) use of amplification, 2) use of oral or total communication, 3) English as the primary language, 4) no visual impairment, 5) ages between 4:0 and 8:11, and 6) phonological awareness difficulty based on the SLP’s or teacher’s evaluation records. A detailed profile for each participating child is shown in Tables 1 and 2.

Ten children were enrolled in preschool or kindergarten, and 10 children were enrolled in first or second grade. Nine children had a mild to moderate hearing loss, nine children had a moderate to severe loss, and two children had a severe to profound loss. Eighteen children wore bilateral behind-the-ear hearing aids and two children wore bilateral cochlear implants. Three children used total communication in a self-contained classroom while the rest of the children used oral communication only in aural and verbal classrooms. In addition to hearing loss and language disorder, two children had a comorbid diagnosis: apraxia of speech (CE6) or Kabuki syndrome (CE9).

All children received speech-language therapy at schools or at a private SLP practice while the study was conducted. The children enrolled in preschool and kindergarten received classroom based phonological awareness instruction. In contrast, the children enrolled in first and second grades did not receive specific phonological awareness instruction, as it was expected that students in these grades would have already mastered most phonological awareness skills and progressed to more difficult decoding skills. The classroom teachers reported that specific phonological awareness instruction for children in first or second grade was addressed mainly during the prior fall semester when a new school year started; general literacy instruction was provided throughout the school year. Study-based interventions for the first and second grade children were provided during the summer or spring semesters.

**Figure 1:**
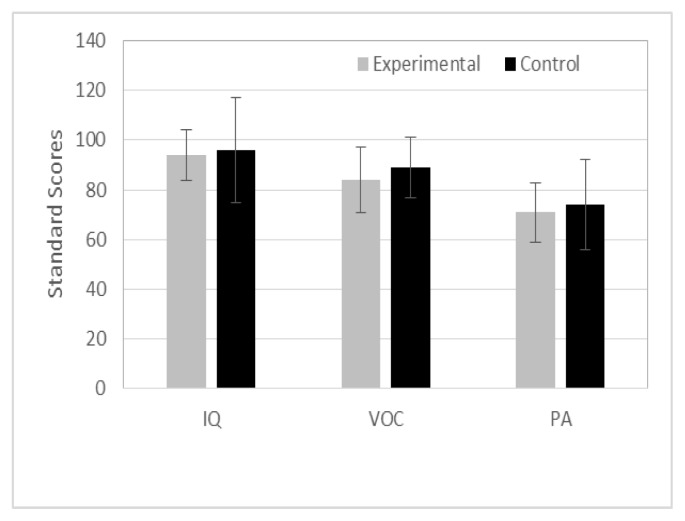
Means and standard deviations of nonverbal IQ, vocabulary (VOC), and phonological awareness skills (PA) between telepractice (experimental) and in-person (control) group.

The three children who used total communication were assigned to the in-person group. The other children were assigned to either the telepractice group or the in-person group. In each group, five children were either preschool or kindergarten students, and five children were in first or second grade. The mean ages for the telepractice group (M= 82 months) and the in-person group (M= 80 months) were not significantly different (t (df = 18) = .264, p = .795). The children were further evaluated in-person via a battery of psychometric tests to ensure that children in each group had similar IQs, vocabulary, and phonological awareness skills. Nonverbal IQ was assessed using the Columbia Mental Maturity Scale-3 (CMMS, Burgemeister, Blum, & Lorge, 2004). The Receptive One-Word Picture Vocabulary Test (ROWPVT-4, Brownell, 2012) was used to test receptive vocabulary. Phonological awareness skills were tested using the Phonological Awareness subtest of the Emerging Literacy & Language Assessment (ELLA, Wigg & Secord, 2004). Figure 1 shows the mean and standard deviations of standard scores of each test for both groups. T-test results for pre-tests revealed that both groups were similar in vocabulary (t (df = 18) = -.760, p = .457), nonverbal IQ (t (df = 18) = −.534, p = .600) and phonological awareness skills (t (df = 18) = −5.16, p = .612).

### PHONOLOGICAL AWARENESS MEASURES

ELLA (Wigg & Secord, 2004) is a phonological awareness test for children aged 4:6 to 9:11. This is a norm and criterion-referenced test, providing standard scores, confidence intervals, percentile ranks, and age equivalents. ELLA is composed of three sections: 1) phonological awareness, 2) sign and symbol recognition, and 3) memory, retrieval, and automaticity. Only the phonological awareness section was used for the current study; this measures the following components of phonological awareness: 1) letter-sound identification, 2) rhyming awareness and production, 3) initial sound identification, 4) blending words, syllables, and sounds, 5) segmenting words, syllables, and sounds, 6) deleting sounds in the initial and final positions of words, and 7) substituting sounds in the initial and final positions of words.

### DEVELOPMENT OF A SUPPLEMENTAL PHONOLOGICAL AWARENESS INTERVENTION PROGRAM

A supplemental phonological awareness intervention program was designed to improve each child’s understanding of how phonemes work together to create words in spoken language, and how phonemes connect to written language. The program design was based on some of the principles discussed by Gillon (2008) and included a variety of activities related to four areas of phonological awareness: rhyme, phoneme identity, syllable-phoneme changes, and speech to print. Similar to the work of Gillon (2008) and previous studies, the program emphasized skills at the phonemic level, (Brady et al., 1994; Brennan & Ireson, 1997; Cary & Verhaeghe, 1994; Lundberg, Frost, & Petersen, 1988), integration of letter-sound knowledge throughout activities, (Cunningham,1990; Hatcher, Hulme, & Ellis, 1994), and incorporation of a variety of activities related to phoneme examination and synthesis (Ayres, 1995; O’Connor et al., 1993; Schneider et al., 1997; Torgesen et al., 1992).

A typical session addressed more than one of the four areas of phonological awareness because research suggests that phonological awareness skills don’t necessarily develop in a linear fashion. The intervention program’s goal was to explicitly expose a child to each of the areas rather than work to a specific accuracy level for each skill. Individual skills were broken down into subskills ranging from easier to more advanced to allow children with varying levels of aptitude an opportunity for exposure to that specific skill. Subskills and exemplars follow, and are listed from basic to more complex.

#### Rhyme

- Identification/Discrimination (e.g., “Do these words rhyme: hat, bat?”)
- Generation (e.g., “What word rhymes with cat?”)
- Judgment (e.g., When provided with a set of at least 3 items, “Which one of these does not rhyme?”)
- Categorization (e.g., Sort word families.)

#### Phoneme Identity

- Initial Position (e.g., “Tell me what sound you hear at the beginning of the word cat.”)
- Final Position (e.g., “Tell me what sound you hear at the end of the word cat.”)

#### Syllable and Phoneme Changes

- Blending/Segmentation
  ○ 2 syllables of familiar compound words (e.g., “Let’s tap out the parts in the word “Hotdog”. “Hot”…”Dog”.)
  ○ 3–4 syllables of familiar words (e.g., “What word do you get when you put these parts together – com…pu…ter?”)
  ○ CVC words (e.g., “What word do you get when you put these sounds together- C—A—T?”
- Deletion
  ○ Whole word or syllable (e.g., “Say the word hotdog. Now, don’t say the word dog. What do you get?”)
  ○ Initial or final phoneme that creates a real word (e.g., “Say the word, cup. Now, don’t say the /k/ sound. What do you get?”)
  ○ Phoneme from an initial word that contained a blend (e.g., “Say the word, stop. Now, don’t say the /t/ sound. What do you get?”)
- Manipulation
  ○ Initial phoneme in a CVC word (e.g., “Say the word call. Now, instead of /k/, say /t/. What do you get?”)
  ○ Final phoneme in a CVC word (e.g., “Say the word pat. Now, instead of /t/, say /s/. What do you get?”)
  ○ 1 phoneme of an initial blend in a word (e.g., “Say the word stop. Now instead of /t/, say /l/. What do you get?”)

Blending and segmentation of phonemes were addressed first. Deletion and manipulation was addressed after comprehension of blending and segmenting was established.

#### Speech to print concepts

- One letter or single words
- Digraphs (e.g., “th”, “sh”)
- Complex connections (e.g., /f/ - “f”, “ph”)
  ○ Each area was addressed in this order:
    ■ Grapheme name and sound (e.g., “This letter, S, makes the /s/ sound.”)
    ■ Initial grapheme/sound in a CV or CVC word (e.g., “I see a letter S at the beginning of the word sick.”)
    ■ Final grapheme/sound in VC or CVC word (e.g., “All these words end in the letter G and have a /g/ sound at the end.”)
    ■ Initial graphemes/sounds in a CCV or CCVC word (e.g., “Sky starts with two sounds, /s/ and /k/. The letters “S” and “K”, make the sounds /sk/.”)

## INTERVENTION

Our phonological awareness intervention for telepractice and in-person groups was provided by a SLP intern and SLP graduate students supervised by certified SLPs. A 30-minute intervention was provided twice a week for 12-weeks. Tables 3 and 4 show phonological awareness skills targeted for each participant of the telepractice or in-person group, respectively. Individualized phonological awareness programs were provided to meet an individual child’s needs; however, overall target goals were similar for most children in either preschool or kindergarten, or in the school-aged group. Target goals of phonological awareness tasks for preschool and kindergarten children focused on rhyming, blending and segmenting words and syllables, phoneme identification, and phoneme blending and segmentation. Grapheme name and sound and initial grapheme and sound in a CV or CVC word were also targeted. Phonological awareness intervention for school-aged children focused on initial and final sound identification, blending and segmenting sounds, deletion of initial and final sounds, and manipulation of initial and final sounds. Initial and final graphemes and sounds in a CVC and blended words were targeted. Typically two goals were addressed in each session. A cyclical goal attack strategy was adopted to address these target goals. For example, for one child in the telepractice group, rhyme identification and rhyme discrimination were targeted during the first week. During the second week, rhyme categorization and two syllable of compound words were addressed. When phoneme blending and the segmentation task were addressed, rhyme identification was readdressed in the following week.

### INTERVENTION PROCEDURE FOR TELEPRACTICE GROUP

A clinician provided telepractice intervention at a university telepractice lab. Ten children assigned to the telepractice group received an individualized phonological intervention using a computer in a classroom, library, or computer lab. Prior to intervention, internet connectivity speeds for the participants and clinicians were tested to ensure adequate connectivity. A minimum of 1.5 Mbps were maintained during intervention. In addition to personal amplification systems (either hearing aids or cochlear implants), the children were equipped with a Hearing Assistive Technology System (HATS) including a Phonak Roger Inspiro Transmitter and Roger X receiver. The HATS allowed the children access to the spoken language of the clinician via direct cable from the computer audio output during intervention in the telepractice group. The HATS helped maintain +10 signal-to-noise ratio (SNR) during telepractice intervention. The telepractice platform selected for the current study was Presencelearning.com. This platform allows a dynamic interaction between a clinician and a client using various games and manipulative hands-on activities.

Before phonological awareness intervention began, a clinician checked the amplification equipment to ensure the HATS was connected appropriately to the computer. Then, a Ling 6 sound test (Ling 1976, 1986) was performed to confirm auditory access to spoken language across the frequency spectrum. This procedure took approximately 5 minutes in each session. Two different phonological skills from the categories listed earlier were targeted in each 30 minute session. After confirming that the auditory signals were appropriate for the child, the first phonological skill was targeted using a variety of activities such as Go Fish, Memory, Bingo, or Picture Sorting for 10 minutes. Another phonological skill was targeted during the next 10 minutes. Exemplars of phonological awareness activities using telepractice included the following:

Rhyme identification exemplars: Three pictures portraying “Dog,” “Cat,” and “Fox,” were shown on the screen. The child was asked to point to or circle the picture of the word that rhymes with “Box” using a mouse.

Initial consonant identification exemplars: Several pictures portraying various initial consonants were shown on the screen. The child was asked to circle pictures starting with the /s/ sound using a mouse.

During the last five minutes in a session, the clinician reviewed the content. For example, “Today, we talked about rhyming. Two words rhyme when the ends of words sound the same.” Also, the clinician probed for possible targets to address next session.

In the telepractice condition, an undergraduate SLP student was present to assist. The assistant wore headphones that were connected to the computer via a Y cord on the audio output, to access the spoken language of the clinician providing the intervention. The assistant was trained on how to login to the telepractice platform and use basic trouble-shooting strategies. When a child had difficulty using the mouse, the assistant helped the child to respond correctly. The assistant also helped the child maintain attention and engage in the telepractice session.

### INTERVENTION PROCEDURE FOR IN-PERSON GROUP

A clinician provided individual phonological awareness intervention to a child in a classroom. Procedures for the in-person group were similar to that of telepractice condition. The children in this group wore a personal amplification system and the HATS including Phonak Roger Inspiro Transmitter and Roger X receiver. The HATS adjusted dynamically to maintain approximately +10 SNR during the in-person intervention. The clinician also wore the HATS transmitter which transmitted the auditory signal. A microphone was clipped at a location at the manufacturer recommended distance (12 cm. from the mouth) to provide enhanced spoken language stimuli during intervention. The clinician first checked the child’s amplification and HATS to ensure the child received good auditory signals. Also, the Ling 6 sound test (Ling, 1976, 1986) was performed to confirm auditory access to spoken language across the frequency spectrum. Then, two goals were addressed, each for 10 minutes. The same materials adopted in telepractice sessions were used for in-person sessions. In addition, commercially available 2- and 3-dimensional educational materials for phonological awareness skills were used. For instance, the telepractice platform allowed for creation of a Memory game to address a rhyming task. For the in-person group, a commercially available Memory game was used for the same task. In addition, 3-dimensional objects were used with the in-person group, especially preschool and kindergarten children. For example, three objects (cake, car, ball) were presented to a child. The child was asked to point to an object that starts with a different sound.

### IMPLEMENTATION FIDELITY

The intervention program was reviewed and supervised by certified SLPs experienced in the treatment of children with hearing loss. Prior to participation, the clinicians were trained by the primary investigators to ensure consistent implementation of the telepractice or in-person interventions, and operation of the telepractice platform. Immediate correction was made by the SLP supervisors if any deviation in procedure occurred. Recorded intervention sessions were randomly selected and reviewed by another SLP supervisor. Implementation procedures were consistent across sessions and all reviewed sessions were conducted as planned.

### MEASURE OF PROGRESS AND RELIABILITY

After 12-weeks of intervention, each child was individually re-tested by a trained member of the research team. This occurred in-person, using the ELLA (Wigg & Secord, 2004). Assessment sessions were recorded using an audio recorder. Raw and standard scores of the ELLA were calculated via the instruction manual. A different member of the research team rescored 10% of assessment tasks. Mean scoring reliability scores were 98%.

### STATISTICAL ANALYSIS

A mixed analysis of variance (ANOVA) was adopted for statistical comparison of standard scores of the ELLA using SPSS (v.20). Standard scores of the ELLA was a dependent variable. Age (two levels: preschool/kindergarten vs. first/second grade) and group (telepractice vs. in-person) were between-subject variables. Test condition (two levels: pretest vs. posttest) was a within-subject variable. A significance level of *p* < 0.05 was adopted. Effect size was calculated using partial eta squared (η^2^*_p_*), interpreting the effect as follows: 0.00–0.09 = negligible, 0.1–0.29 = small, 0.30–0.49 = moderate, and 0.5 and greater = large (Rosenthal & Rosnow, 1984).

## RESULTS

**Figure 2:**
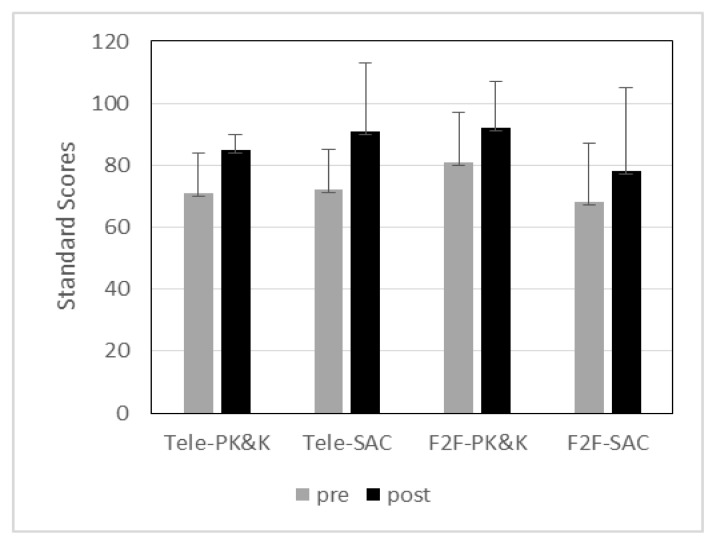
Means and standard deviations of phonological awareness skills between pre- and post-tests between telepractice (experimental) and in-person (control) group.

Figure 2 shows the means and standard deviations of standard scores of ELLA in the four groups (telepractice group with preschoolers/kindergarteners, telepractice group with first/second grader, in-person group with preschoolers/kindergarteners, in-person group with first/second graders) for both pre- and post-tests. The preschool/kindergarten children in the telepractice group received an average of 71 (SD =13) for the pre-test, which fell below average ranges, but their scores improved to an average of 85 (SD = 6.0) for the post-test, which was within average ranges. Similarly, the school-aged children in the telepractice group obtained an average of 72 (SD = 13), and their scores also improved to an average of 91 (SD = 21) for the post-test. Their standard scores were within average ranges for the post-test whereas they fell below average ranges during pre-test. For the in-person group, the preschool/kindergarten children received an average of 81 (SD = 16) for the pre-test, which was within average ranges. They also showed an improvement on phonological awareness skills for the post-test, with an average score of 92 (SD = 16). The first and second grade children in the in-person group received an average of 69 (SD = 19) for the pre-test was whereas they received an average of 78 with a relatively greater variance (SD 27).

A mixed ANOVA revealed no significant three-way interaction for test condition * group * age (*F* (1, 16) = .389, *p* = .542, η2p= .024). There were no two-way interactions for test condition * group (*F* (1, 16) = 1.21, *p* = .288, η2p= .07), test condition * age (*F* (1, 16) = .134, *p* = .719, η2p= .008), as well as age * group (*F* (1, 16) = 1.31, *p* = .268, η2p= .07). There was no main effect for group either (*F* (1, 16) = .420, *p* = .526, η2p= .03). However, there was a significant main effect for test condition (*F* (1, 16) = 25.97, *p* < .001, η^2^*_p_*= .62). The significant main effect for test condition with a larger effect size suggested that post-test scores were significantly higher than the pre-test scores in all four groups.

**Figure 3:**
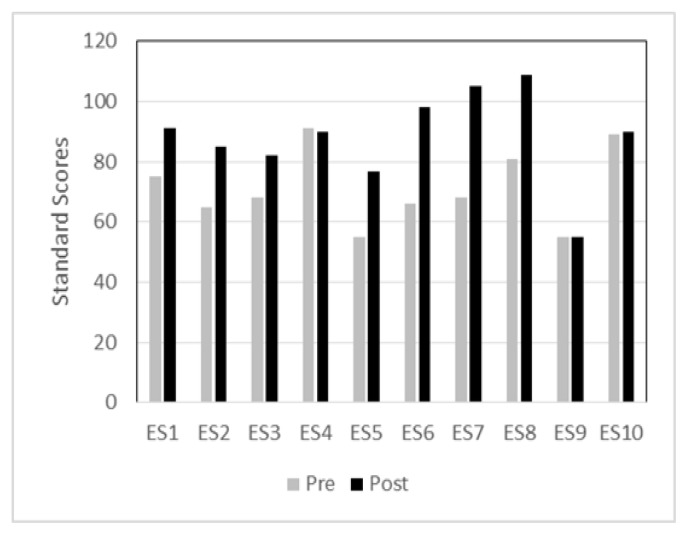
Individual standard scores of pre- and post-tests in the telepractice group.

Figure 3 shows individual standard scores of the ELLA in the telepractice group. Standard scores of only two children were within one standard deviation below the mean for the pre-test whereas those of the other children fell below average ranges. After the 12-week phonological intervention, 7 of the 10 children showed improved phonological awareness skills for the post-test as compared to the pre-test. Among the three children who did not show improvement on phonological awareness skills, one child had a severe-profound hearing loss bilaterally and used bilateral cochlear implants and the other two children were 8-year-olds who demonstrated difficulty in manipulating initial and final segments (e.g., changing the last sound in the word ‘bit’ to /g/).

**Figure 4:**
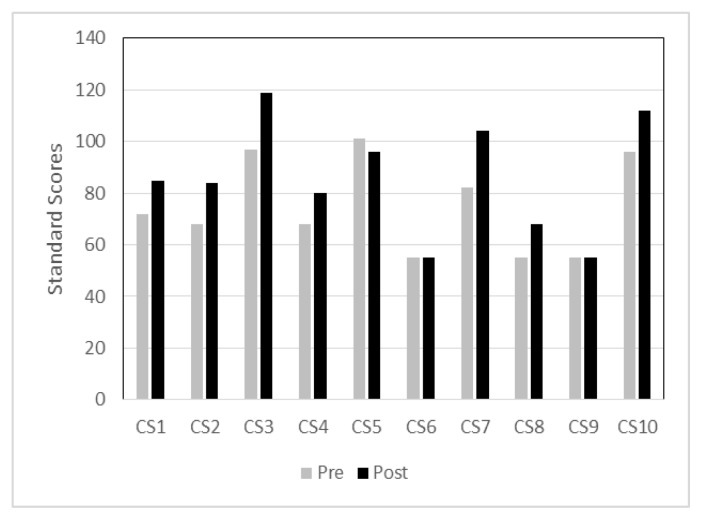
Individual standard scores of pre- and post-tests in the in-person group.

Figure 4 shows individual standard scores of ELLA in the in-person group. Standard scores of three children were within one standard deviation below the mean while the other children’s scores fell below average ranges during pre-test. After the 12-week intervention, 7 of the 10 children showed improved phonological awareness skills during the post-test as compared to the pre-test. Two children showed no improvement between pre- and post-tests, and one child demonstrated a slight decrease on the post-test. Of the three children who did not show improvement or demonstrated a slight decrease on the post-test, two of the three children had a severe-profound hearing loss with bilateral cochlear implants or a severe language delay due to a comorbid diagnosis. The third child had a mild hearing loss without a diagnosed language disorder.

## DISCUSSION

### THE FEASIBILITY OF TELEPRACTICE SERVICE DELIVERY MODEL FOR CHILDREN WITH HEARING LOSS

The primary focus of the current study was to examine the feasibility of telepractice service delivery model for young children with hearing loss, a possibility that has been minimally researched. One study (Behl et al., 2013) was conducted in an early intervention setting. However, it was difficult to examine the effect of direct intervention with infants and toddlers with hearing loss via telepractice because the direct service recipients were mainly parents or caregivers of the children. Only one study (Constantinescu et al., 2014) provided a direct intervention to children with hearing loss; their mean age was 2:4 years.

It is important to examine the feasibility of telepractice service delivery for children with hearing loss, as there are too few qualified service providers to meet the needs of these children (Besculides, Saltzman, Ireys, & White, 2010; Houston, Munoz & Bradham, 2011; Houston & Stredler-Brown, 2012; Joint Committee on Infant Hearing, 2007). In the current study, 14 of the 20 participants demonstrated improvement after a 12-week intervention. There was no significant difference between the two groups’ performance on the post-intervention measure, suggesting that the telepractice service delivery model was as effective as the in-person intervention. The findings of the current study were consistent with those of Grogan-Johnson and colleagues (Gabel et al., 2013; Grogan-Johnson et al., 2010; 2011; 2013) wherein speech and language interventions via telepractice were as effective as in-person treatment for non-hearing impaired children with speech and language disorders. Our findings were also consistent with Constantinescu et al. (2014) wherein the outcomes of telepractice service were similar to outcomes of in-person methods for children with hearing loss.

The results of the current study suggest that a telepractice service delivery model that incorporates an adequate frequency modulate system, is feasible for use with young children beyond 2:4 years of age with hearing loss. One of the important tasks for implementing the telepractice service delivery model is to identify appropriate candidates.

### THE FEASIBILITY OF PHONOLOGICAL AWARENESS INTERVENTION VIA TELEPRACTICE

Previous studies have evaluated the use telepractice to improve children’s articulation or language skills The current study is the first study to examine the feasibility of phonological awareness intervention via telepractice. This is an important area of inquiry because children with hearing loss (regardless of degrees of hearing loss) demonstrate delayed phonological awareness, despite having received in-class phonological awareness instruction their classroom teachers (Kyle & Harris, 2011; Miller, 1997; Moeller et al., 2007; Most, Aram, & Andorn, 2006; Sterne & Goswami, 2000). The current study suggests that a supplemental phonological awareness instruction could be a viable option to support the development of phonological awareness these children. This also may result in improved reading outcomes.

### INDIVIDUAL VARIANCES

Studies of phonological awareness intervention for children with hearing loss (Miller et al., 2013; Smith & Wang, 2010; Syverud et al., 2009), though limited in number, have suggested that explicit phonological awareness instruction leads to increased skills. The present study extends earlier work and provides information based on a larger group of participants. Similar to the previous work, we found that the majority of children with hearing loss (14 out of 20) improved their phonological awareness skills after a 12-week intervention.

Studies have shown considerable variability in phonological awareness skills of children with hearing loss (Dillon et al, 2012; James et al, 2008; Webb & Lederberg, 2014). Some children achieve scores in the normal range while others lag behind, with the variability attributed to differences such as type of amplification, age at audiological intervention, communication modes, educational environments, and formal instruction in reading. We also found individual differences in the treatment effects of phonological awareness intervention. Among the six children who did not show improvement during intervention, two participants were the oldest children (8-year-olds) whose difficulties were mainly limited to initial and final sound substitutions. These highest level tasks did not improve even after a 12-week phonological awareness intervention.

Two other children who did not show improvement were children with severe-to-profound hearing loss who were equipped with bilateral cochlear implants. Therefore, our results suggest the degree of hearing loss, as well as the type of task may be factors affecting acquisition of phonological awareness skills. Further studies are warranted to verify our findings.

One child who showed no improvement on standard scores between pre-and post-tests, had a nonverbal IQ that was 1.5 standard deviations below the mean. His lower nonverbal IQ may have affected his acquisition of phonological awareness skills. Another child with a similar nonverbal IQ showed improvement after intervention. Thus, it is difficult to argue that his acquisition of phonological awareness skills was affected by his IQ. This child was also one of the oldest children in the study. The age may also effect the results similar to the other oldest children. Finally, the latter child demonstrated no specific factors that explain an inability to improve phonological awareness skills. However, this child’s raw scores on all subtests of the post-test were still increased as compared to those of pre-test. Therefore, our findings suggest that most children with hearing loss may receive benefits from phonological awareness intervention; however, maximum benefit of phonological awareness intervention may be limited to children with severe to profound hearing loss or children with limited room for improvement.

Previous studies of phonological awareness intervention in children with hearing loss examined only young children. For example, Smith and Wang (2010) and Werfel and Schuele (2014) examined 4-year-olds whereas Miller et al. (2013) included children aged 3:8 to 5:11. Only Syverude et al. (2009) examined a first grade child with hearing loss. Thus, further studies are warranted to determine whether phonological awareness intervention can be effective for older children, as well as those with severe to profound hearing loss.

## CONCLUSIONS AND STUDY LIMITATIONS

The current study conducted a larger scale study of intervention via telepractice than prior studies, therefore providing more compelling empirical evidence regarding the feasibility of the telepractice service delivery model for SLP services. Based on our findings, the telepractice service delivery model may be equivalent to the traditional method. The current study also provided empirical evidence to support the use of telepractice for improving phonological awareness skills in children with hearing loss.

However, the current study had several limitations for future studies to take into account. First, the current study employed a small number of children in wide age ranges. Although 10 children in each group was larger than previous studies of phonological awareness intervention, there were only had five children in each age group of each service delivery model. Since phonological awareness skills are very different in each age group, future studies may narrow age ranges to verify our findings.

Second, since the primary purpose of the current study was to examine the feasibility of phonological awareness intervention via telepractice across a wide age range, intervention included many phonological awareness tasks. Future studies may examine one or two phonological awareness tasks.

Third, while the current study was conducted, phonological awareness instruction was provided in the classroom by classroom teachers, in particular for preschool and kindergarten children. Thus, it is difficult to argue that improvement of phonological awareness skills in participating children was solely attributed to our supplemental phonological awareness intervention via telepractice. However, we also found that improvement among first and second grade children as a group although direct classroom phonological instruction was minimal. Instead, the first and second grade children in telepractice group showed the largest improvement among the four groups. Since phonological awareness instruction is required in schools in the US, it is difficult to control the effect of classroom phonological awareness instruction when an intervention study is implemented with school aged children. A recent study examining the effect of phonological awareness instruction also had the same limitation (Goldstein et al., 2017). Thus, further studies examining phonological awareness intervention via telepractice may consider how to control the classroom phonological intervention effect. A related limitation is that the study did not employ a control group that received no study-based intervention, to ensure that the gains seen in phonological awareness did not occur due to development/maturation, or the sole effect of classroom phonological instruction.

Finally, due to the nature of telepractice and in-person service delivery models, the materials used in both groups were not identical. For example, the telepractice platform used in the current study allowed a clinician to create a Bingo game for the telepractice group. An equivalent commercially available Bingo game was used for the in-person group. Objects were included in the in-person group, but not the telepractice group. The effect of different service delivery models needs to be carefully examined in future studies. In short, more well-controlled experimental studies are warranted to investigate the effect of the telepractice service delivery model in the future.

## Figures and Tables

**Table 1:** Profiles for Telepractice Group Participants

	ES1	ES2	ES3	ES4	ES5	ES6	ES7	ES8	ES9	ES10
Age	6:0	5:6	6:5	6:5	6:7	6:8	7:4	7:0	8:0	8:8
Grade	PreK	K	K	K	K	1ST	1ST	1ST	2ND	2ND
Gender	M	F	F	F	F	M	F	F	M	M
Mode	Oral	Oral	Oral	Oral	Oral	Oral	Oral	Oral	Oral	Oral
Home Language	SEB	SEB	SEB	ME	ME	ME	ME	ME	ME	SEB
Degree of HL (Left)	Mild-sev	Mod-mod sev	Mod-sev	Profound	Mild-mod	Mild-mod	Mild-mod	Mild-mod	Profound	Mod-mod sev
Degree of HL (Right)	Mild-sev	Mod-mod sev	Mod-sev	Profound	Mild-mod	Mid-mod	Mod	Mild-mod	Sev-mild	Mod-mod sev
Uni/Bi	BI	BI	BI	BI	BI	BI	BI	BI	BI	BI
Amp Type	HA	HA	HA	CI	HA	HA	HA	HA	HA	HA
Age ID	0:4	0:0	0:0	0:0	3:0	2:7	1:9	0:0	0:11	0:0
Age Amp	0:7	0:6	1:6	3:0(R) 4:0(L)	5:4	3:0	1:9	3:6	5:6	2:11
Age Served	0:4	0:5	1:6	0:3	6:8	3:4	1:9	3:9	1:0	2:10
ELLA-Pre	75	65	68	91	<55	66	68	81	<55	89
VOCAB	91	76	72	66	71	108	83	102	87	86
IQ	89	89	96	106	106	78	91	90	89	111

**Table 2:** Profiles for In-person Group Participants

	CS1	CS2	CS3	CS4	CS5	CS6	CS7	CS8	CS9	CS10
Age	4:6	5:5	4:11	5:8	5:6	7:9	7:7	8:5	8:11	8:4
Grade	PreK	PreK	PreK	K	K	1st	1st	2nd	2nd	2nd
Gender	M	M	M	M	F	M	M	M	M	M
Mode	Oral	Oral	Oral	Oral	Total	Total	Oral	Total	Oral	Oral
Home Language	ME	SEB	SEB	SEB	ME	ME	ME	ME	ME	ME
Degree of HL (Left)	Mild	Mild-mod	Mild-mod	Mod sev	Mod-mod sev	Sev-Profound	Mod-sev	Mod	Sev	Mild-mod
Degree of HL (Right)	NA	Mild-mod	Mod	Mod sev	Mod-mod sev	Profound	Mild-mod	Mod	Mild-mod sev	Mild-mod
Uni/Bi	UNI	BI	BI	BI	BI	BI	BI	BI	BI	BI
Amp Type	HA	HA	HA	HA	HA	CI	HA	HA	HA	HA
Age ID	0:0	4:5	0:0	0:1	3:3	0:11	0:1	0:0	0:0	5:0
Age Amp	0:3	4:7	0:4	0:9	3:5	2:3	0:5	0:6	0:3	5:0
Age Served	0:4	4:8	0:5	0:3	4:1	1:0	NA	0:8	1:0	5:3
ELLA-Pre	72	68	97	68	101	<55	82	<55	<55	96
Vocab	101	86	92	88	107	87	110	72	75	80
IQ	77	115	106	92	97	111	113	74	72	124

*Note.* Home Language: SEB = Spanish-English bilingual, ME = Monolingual English; Age ID: Age identified; Age Amp: Age amplified; Age Served: Age enrolled in the Regional Day School Program for the Deaf

**Table 3:** Phonological Awareness Skills Targeted for Telepractice Group Participants

Level of Difficulty	Target category	Target goals	ES	ES	ES	ES	ES	ES	ES	ES	ES	ES
			1	2	3	4	5	6	7	8	9	10
Most difficult -------------------------------Least difficult	Rhyming	Rhyme identification	X	X	X	X	X					
		Rhyme discrimination	X	X	X	X	X					
		Rhyme judgement	X	X	X	X	X					
		Rhyme categorization	X	X	X	X	X					
	Syllable/Words	2 syllable of compound words	X	X	X	X	X					
	Blending Segmentation	3–4 syllable of familiar words	X	X	X	X	X					
	Syllable/Words Deletion	Whole word or syllable deletion	X	X	X	X	X					
	Phoneme identification	Initial or final phoneme identification	X	X	X	X	X	X	X	X	X	
	Phoneme blending/segmentation	Phoneme blending/segmentation	X	X	X	X	X	X	X	X	X	
	Rhyming	Rhyme generation						X	X	X	X	X
	Phoneme deletion	Initial or final phoneme deletion						X	X	X	X	X
		A phoneme deletion from initial consonant blends								X	X	X
	Phoneme manipulation	Initial phoneme manipulation in a CVC word								X	X	X
		Final phoneme manipulation in a CVC word								X	X	X
		Initial phoneme manipulation in consonant blends								X	X	X

**Table 4:** Phonological Awareness Skills Targeted In-person Group Participants

Level of Difficulty	Target category	Target goals	CS	CS	CS	CS	CS	CS	CS	CS	CS	CS
			1	2	3	4	5	6	7	8	9	10
Most difficult -------------------------------Least difficult	Rhyming	Rhyme identification	X	X	X	X	X					
		Rhyme discrimination	X	X	X	X	X					
		Rhyme judgement	X	X	X	X	X					
		Rhyme categorization	X	X	X	X	X					
	Syllable/Words	2 syllable of compound words	X	X	X	X	X					
	Blending Segmentation	3–4 syllable of familiar words	X	X	X	X	X					
	Syllable/Words Deletion	Whole word or syllable deletion	X	X	X	X	X					
	Phoneme identification	Initial or final phoneme identification		X	X	X	X	X			X	
	Phoneme blending/segmentation	Phoneme blending/segmentation		X	X	X	X	X	X	X	X	
	Rhyming	Rhyme generation						X	X	X	X	X
	Phoneme deletion	Initial or final phoneme deletion						X	X	X	X	X
		A phoneme deletion from initial consonant blends							X	X		X
	Phoneme manipulation	Initial phoneme manipulation in a CVC word							X	X		X
		Final phoneme manipulation in a CVC word							X	X		X
		Initial phoneme manipulation in consonant blends							X	X		X
